# Targeting post-translational modifications of Foxp3: a new paradigm for regulatory T cell-specific therapy

**DOI:** 10.3389/fimmu.2023.1280741

**Published:** 2023-10-23

**Authors:** Farooq Riaz, Zhihui Huang, Fan Pan

**Affiliations:** Shenzhen Institute of Advanced Technology (SIAT), Chinese Academy of Sciences (CAS), Shenzhen, China

**Keywords:** regulatory T cells, Foxp3, immunotherapy, tumor microenvironment, anti-tumor immunity, autoimmune diseases, post-translational modifications

## Abstract

A healthy immune system is pivotal for the hosts to resist external pathogens and maintain homeostasis; however, the immunosuppressive tumor microenvironment (TME) damages the anti-tumor immunity and promotes tumor progression, invasion, and metastasis. Recently, many studies have found that Foxp3+ regulatory T (Treg) cells are the major immunosuppressive cells that facilitate the formation of TME by promoting the development of various tumor-associated cells and suppressing the activity of effector immune cells. Considering the role of Tregs in tumor progression, it is pivotal to identify new therapeutic drugs to target and deplete Tregs in tumors. Although several studies have developed strategies for targeted deletion of Treg to reduce the TME and support the accumulation of effector T cells in tumors, Treg-targeted therapy systematically affects the Treg population and may lead to the progression of autoimmune diseases. It has been understood that, nevertheless, in disease conditions, Foxp3 undergoes several definite post-translational modifications (PTMs), including acetylation, glycosylation, phosphorylation, ubiquitylation, and methylation. These PTMs not only elevate or mitigate the transcriptional activity of Foxp3 but also affect the stability and immunosuppressive function of Tregs. Various studies have shown that pharmacological targeting of enzymes involved in PTMs can significantly influence the PTMs of Foxp3; thus, it may influence the progression of cancers and/or autoimmune diseases. Overall, this review will help researchers to understand the advances in the immune-suppressive mechanisms of Tregs, the post-translational regulations of Foxp3, and the potential therapeutic targets and strategies to target the Tregs in TME to improve anti-tumor immunity.

## Introduction

During the formation and growth of the tumor, immune cells not only suppress the tumor but also shape the immunogenicity of cancer cells in three distinct phases of cancer immunoediting, including the elimination phase (tumor cells show strong immunogenicity), equilibrium phase (tumor cells managed to  survive to exhibit low immunogenicity), and escape phase (tumor cells escape immune recognition) (1). In the tumor microenvironment (TME), there is a continuous interaction between tumor cells and immune cells. Exploring the intrinsic mechanism demonstrating this interaction between the tumor and the immune cells is essential to find targeted therapeutic strategies. Meanwhile, several tumor-associated cell types, including tumor-associated macrophages (TAMs), tolerogenic dendritic cells (DCs), and myeloid-derived suppressive cells (MDSCs) have been seen in the TME, which play different functions compared to their normal cell types (2, 3). Besides, TME disrupts immune cell function by manipulating glucose metabolism, lactic acid metabolism, hypoxia, and tumor-associated cells. However, the involvement of various tumor-associated cell types and the interaction of immune and cancer cells in TME lowers the efficiency of a targeted drug to treat a specific tumor.

In the TME, several novel CD4^+^ T subsets have been identified through research in recent years. Among these subsets, one of the most important milestone is the discovery of regulatory T (Treg) cells, which are crucial for the immune system to deal with self-antigen and maintain homeostasis by suppressing the immune response to foreign pathogens or self-tissue response (4, 5). Tregs are characterized by constitutive expression of the transcription factor forkhead box protein P3 (Foxp3) (6). Foxp3 is a member of the forkhead box (Fox) family, subfamily P. This subfamily has four members in mammals, namely Foxp1-4 (7). Foxp3 is the most conserved across mammals. For instance, the similarity between the amino acid sequences of human and mouse Foxp3 is 91% (8). Foxp3, an oligomeric molecule and a critical transcription factor, comprises four key domains: an N-terminal domain (amino acids 1-97) for transcriptional control, a central zinc-finger domain (amino acids 197-222) involved in interactions and possibly oligomer formation, a leucine-zipper domain (amino acids 239-260) crucial for protein interactions, and a C-terminal forkhead domain (amino acids 337-423) which allows Foxp3 to bind specific DNA sequences, regulating gene expression. Mutations in these domains can lead to autoimmune disorders like IPEX syndrome, highlighting their collaborative role in the function of Tregs (9–12). The Foxp3 promoter is activated by a range of transcription factors, including NFAT and AP-1, in response to TCR signaling and co-stimulation pathways (4). Additionally, Forkhead box protein O (FOXO) proteins, specifically FOXO1 and FOXO3, have been observed binding to the Foxp3 promoter and other regulatory elements of the Foxp3 gene (13). CREB (cAMP response element binding protein)-activating transcription factor 1 (ATF1) complexes also contribute to Foxp3 promoter activation (14). Notably, Foxp3 promoter exhibits modest trans-activating potential, with its regulation heavily dependent on conserved enhancer regions. These interactions play a crucial role in the regulation of Foxp3 gene expression (4).

Foxp3 plays a central role in the maturation and immunosuppression function of Tregs by regulating the gene expression of the target gene through direct interaction and supporting the immunosuppressive microenvironment by expressing suppressive molecules of Tregs (6, 15). It is evident that loss or gain of Foxp3 also minimizes or maximizes the immunosuppression function of Tregs, which ultimately attenuates severe autoimmune diseases or tumor development (16). Besides the genetic manipulation of Foxp3 to regulate its functions, transcriptional and post-translational modifications (PTM) have also been recognized to regulate Foxp3 function (15, 17). The highly specific and versatile PTMs provide new insights into Foxp3 functions and therapeutic approaches. In this review, we will discuss PTMs of Foxp3 and emphasize the function and suppressive mechanisms of Tregs in tumors. Moreover, we will also highlight the potential of Treg-targeted immunotherapy.

## Immunosuppressive mechanism of Treg in the tumor microenvironment

**Figure 1:**
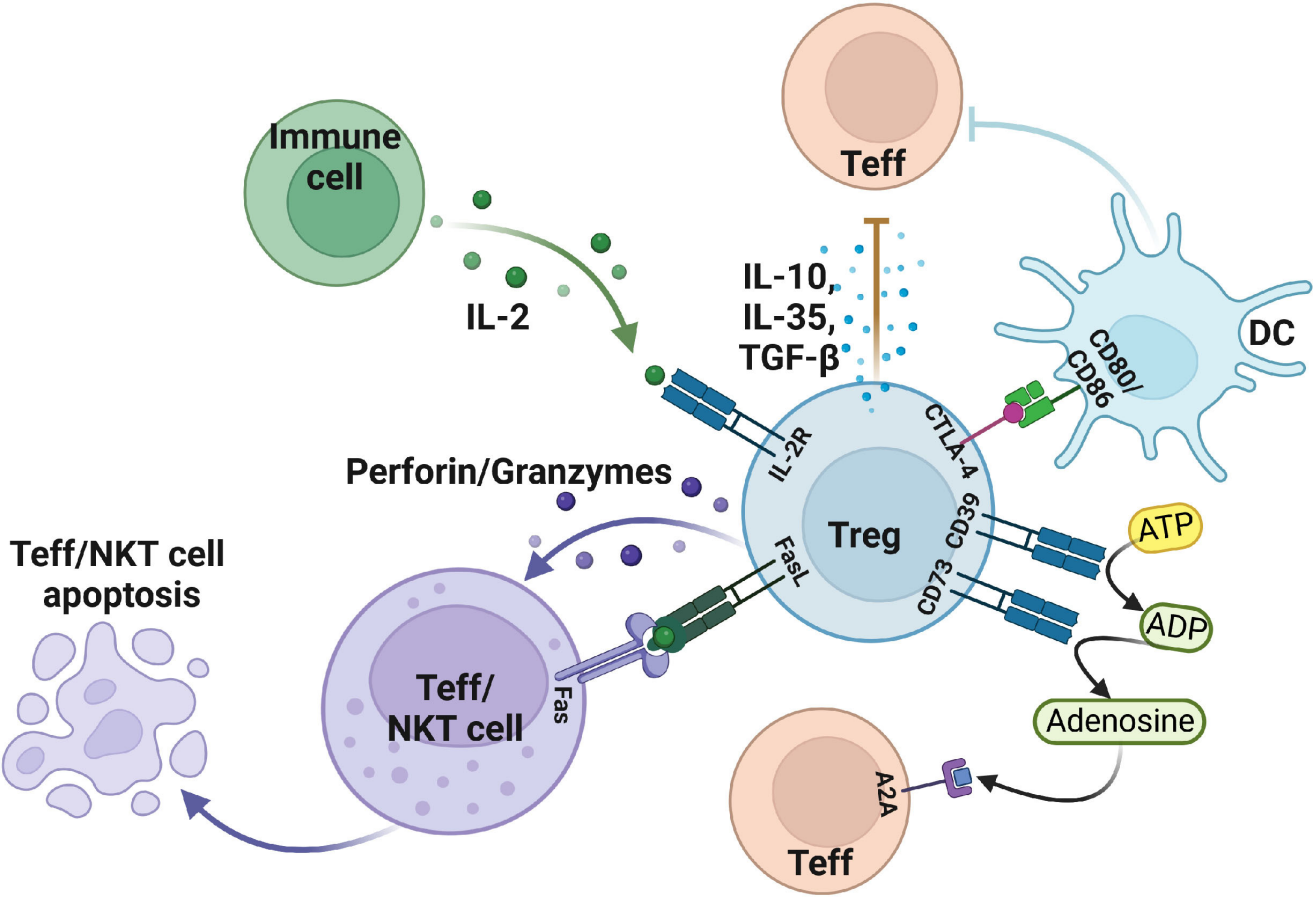
The suppressive function of Treg in the tumor microenvironment. Treg cells induce apoptosis of effector T and NKT cells by expressing FasL, granzyme, perforin, and IL-2R. In addition, Treg also secretes IL-10, TGF-β, IL-35, and other cytokines to make effector T cells less functional. Moreover, Treg cells mediate DC, and effector T cell status by expressing negative regulatory receptors. Figure was created with BioRender.com.

CD4^+^CD25^+^Foxp3^+^Tregs are the immunosuppressive cells that play pivotal roles in maintaining immune homeostasis. Tregs maintain their function through a variety of inhibitory pathways. However, Tregs not only play an active role in healthy humans but also plays a side effect in patients with tumors or inflammation by inhibiting the effector T, DC, and NK cells, thereby promoting the occurrence and development of tumors in patients, which results in poor prognosis (18). It has been illustrated that Granzyme B is not expressed in nTreg but is highly expressed in 5%-30% of tumor infiltrating Treg (19). Tumor-derived Tregs mediate immunosuppressive effects by expressing granzyme B and perforin to induce apoptosis of effector T and NK cells. At the same time, Tregs can mediate apoptosis by expressing FasL-Fas (**Figure 1**) (19–22). Interleukin 2 (IL-2) is necessary for the suppressive function and the survival of Tregs (23). Tregs produce a low amount of IL-2 cytokine but highly express IL-2R, of which CD25 is the α chain of IL-2R. IL-2 capture is dispensable for CD4^+^ T cells, but IL-2 deprivation can limit the activation of CD8^+^ T cells, which require IL-2 cytokine to activate and maintain their cytotoxic effect. Thus, large amounts of IL-2R in Tregs deprive effector T activation and enable the apoptosis-mediated inhibition effect of Tregs (24, 25).

In addition to the secretion of suppressive cytokines, Treg cells can exert suppressive functions through cell surface receptors. Treg cells generally express inhibitory cytotoxic T lymphocyte-associated protein 4 (CTLA-4) on the cell surface, whereas Tconv can also express it. CTLA-4 has two functions in Tregs: 1) protect against autoimmune diseases; 2) avoid excessive inflammatory responses. CTLA4^+^ Tregs can downregulate the CD80/CD86 in DC cells to inhibit their maturation which can eventually reduce the activation of effector T cells (26, 27). On the other hand, CTLA-4 interacts with CD80/CD86 and induces DC to express indoleamine 2,3 dioxygenase (IDO) catabolize tryptophan which can inhibit the response of effector T cells *in vivo (* 28). Some studies found that DCs from interferon γ (IFN-γ)-receptor-deficient mice don’t affect the induction of IDO, indicating that IFN-γ is not necessary for Tregs (29).

The enzymatic activities of the CD39 and CD73 receptors also mediate Treg immunosuppression. Mechanistically, CD39/CD73 turns a pro-inflammatory extracellular microenvironment into an anti-inflammatory microenvironment through ADP/ATP to AMP and then AMP to adenosine, respectively. This adenosine binds to the adenosine receptor A2A and activates an immune regulatory pathway to mediate the differentiation of Th1 and Th2 (30, 31). Treg cells were discovered to transform DCs into a functional and phenotypically tolerogenic form by producing IL-10 and TGF-β (32). Tumor-derived Tregs induce the generation of tolerogenic DCs, which finally reduce the expression of co-stimulatory receptors and the secretion of pro-inflammatory cytokines, including IL-6, IL-12, and TGF-β. Therefore, tolerogenic DCs can’t effectively stimulate the other effector T cells, thus suggesting another Treg-mediated form of immunosuppression (33–36).

Similarly, Treg cells can inhibit pro-inflammatory macrophages and guide macrophages to differentiate into anti-inflammatory cytokine phenotypes through immunomodulatory effects. TGF-β and IL-10 are the key inhibitory cytokines in this process (37). In addition, tumors overexpressing IDO inhibit the recruitment and functional activation of myeloid-derived suppressor cells (MDSCs) after the systemic deletion of Foxp3^+^ Treg cells (38). Aryl-hydrocarbon receptor (AHR) plays a distinct role in immune cells, particularly in the development of Tregs in multiple diseases (39). Some research reports that tryptophan catabolism by IDO/TDO acts as AHR ligands and promotes immunosuppressive effects in different tumors. The immunosuppressive effects mediated by IDO-Kyn-AHR depend on the interaction of Treg and tumor-associated macrophages (TAMs). Selective inhibition of AHR combined with PD-1 therapy can effectively delay tumor growth (40). Collectively, the immunosuppressive mechanism of Tregs in TME is highly cross-linked and comprehensive, which further needs investigation.

## Post-translational modifications of Foxp3

Post-translational modifications (PTMs) refer to cellular processes that manipulate the expression of a specific protein and significantly impact the overall characteristics of that particular protein, including the turnover, interaction, localization, etc. (41). Consequently, Foxp3 is also highly regulated by numerous processes involving PTMs, including acetylation, glycosylation, phosphorylation, ubiquitylation, and methylation (39). These PTMs of Foxp3 can restrict the function of Tregs in maintaining the immunosuppressive microenvironment (42). In this part, we will discuss recent studies highlighting the role of these PTMs on Foxp3 stability and function.

## Acetylation modification of Foxp3

**Figure 2:**
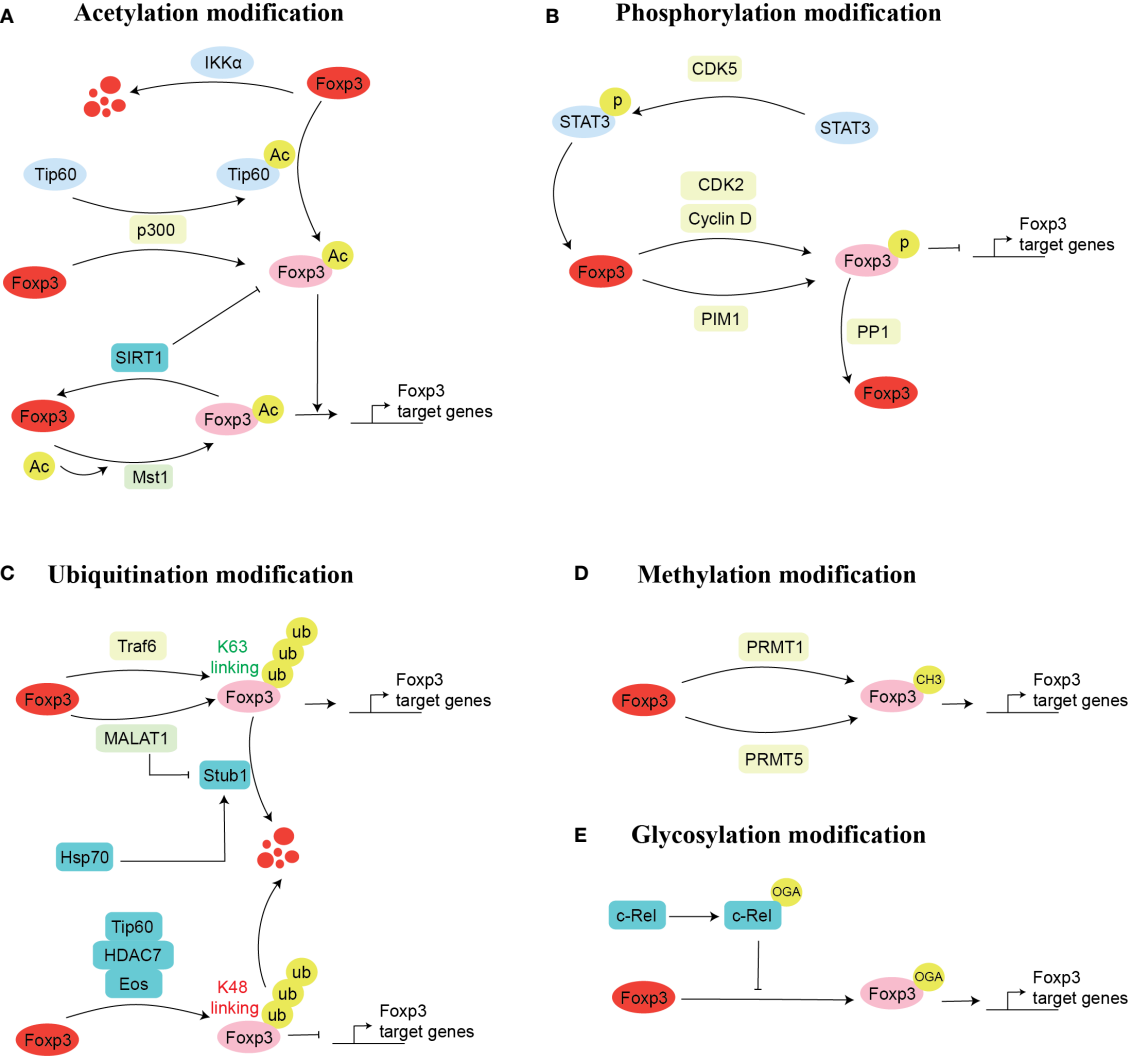
Mechanism of Foxp3 post-transcriptional modification. There are five possible ways for the post-transcriptional modifications of Foxp3. These are acetylation **(A)**, phosphorylation **(B)**, ubiquitination **(C)**, methylation **(D)**, and glycosylation **(E)**. Here, we mentioned the enzymes and proteins and their cellular processes involved in post-transcriptional modifications of Foxp3. Acetylation modification: acetylation (Tip60, p300), deacetylation (SIRT1), promotes acetylation (p300) and inhibits acetylation (Mst1); Glycosylation modification: O-GlcNAcylation (c-Rel); Phosphorylation modification: phosphorylation (CDK2, PIM1, PIM2), dephosphorylation (PPP1); Ubiquitination modification: ubiquitination (TRAF6, Stub1), inhibits ubiquitination (MALAT1); Methylation modification: methylation (PRMT1, PRMT5).

Acetylation is an important process that regulates the expression of Foxp3 (**Figure 2A**). In this process, the coordination of histone deacetylases (HDACs) and histone acetyltransferases (HTAs) introduces or removes an acetyl group to modify a protein (43). HTAs can stabilize protein structure that binds to the target gene to regulate its expression, while HDACs overturn this process (44). Acetylation has been well studied to influence the PTMs of Foxp3. It has been shown that Foxp3 is more stable under the influence of acetylation compared to deacetylation because the acetylation of Foxp3 hinders its degradation. Briefly, it was illustrated that proteasome-mediated ubiquitylation degradation is impaired during the acetylation of lysine residues, which allows the binding of Foxp3 to the chromatin, thus regulating the expression of the downstream gene (45–47).

The long-term imbalance of Treg/Th17 causes multiple cancers and chronic inflammatory diseases, including cancer, metabolic diseases, and autoimmunity (48–51). Numerous acetylases, *i.e.*, Tip60 (Histone acetyltransferase KAT5) (52), CBP/p300 (p300-CBP coactivator family) (53), and MST1 (Macrophage Stimulating 1) (54), have been found to modulate the Foxp3 acetylation in different ways, thus regulating the suppressive function of Tregs. Mechanistically, in rheumatoid arthritis (RA), naïve CD4^+^CD45RO^-^ T produces fewer Foxp3 proteins, which results in the differentiation of naïve T cells into Th17 commitment, while leads to fewer Treg populations, which affects the Th17/Treg balance (52). Detailed molecular mechanism indicate that T cells in RA reduce their ability to produce enough histone acetyltransferase Tip60, leading to a decreased Foxp3 acetylation (52). The complex of Foxp3:Tip60 and Foxp3:HDAC7, and Foxp3:HDAC9 is necessary for the repression by Foxp3 (55). Foxp3 mutation also affects the function of Tregs. P.A384T IPEX mutation in patient-derived Treg cells has been associated with a decreased suppressive function of Tregs. Still, it maintains the ability of Foxp3 to suppress the production of inflammatory cytokines (56). This phenomenon is due to the restriction of Foxp3^A384T^ binding with Tip60 (56). In addition, allosteric modifiers can enhance the Foxp3-Tip60 interaction and the activity of Foxp3 (56).

CBP, a p300 paralog, is crucial in regulating the differentiation of Tregs (57) and the expression of Foxp3 (53). CBP/p300 is closely related to acetyltransferase and transcriptional coactivators of Foxp3. Evidence indicates that Treg-specific deletion of CBP or p300 develops minimal autoimmune disease. In contrast, Treg-specific deletion of both CBP and p300 creates serious consequences and the death of mice in 3 to 4 weeks (53). However, using Garcinol, a natural p300 inhibitor, reduces the suppressive function of Treg cells and improves anti-tumor immunity (58). In addition, some studies have found that p300 activates Tip60 through acetylation, which acetylates Foxp3 and maintains its function and stability. At the same time, Foxp3 can also be directly acetylated by interaction with p300, which can avoid proteasome-mediated Foxp3 degradation (47).

The histone deacetylases (HDACs) can negatively regulate the Foxp3 expression and Treg function (59). Inhibition of HDACs elevates and stabilizes the expression of Foxp3 and sustains the suppressive function of Tregs (59–61). Recent data demonstrated that the acetylation of Foxp3 can be reversed by class III HDAC SIRT1 (Sirtuin1) (62). Briefly, using the major histocompatibility complex (MHC)-mismatched cardiac allografts model, researchers found that targeted deletion of SIRT1 in CD4^+^T cells or Treg cells extends the survival of mice. This demonstrated that SIRT1 could negatively regulate the Treg function (62). Meanwhile, in abdominal aortic aneurysms patients and Hashimoto’s thyroiditis patients, SIRT1 is highly expressed in infiltrated CD4^+^ T cells, and the frequency of Treg is significantly decreased in these patients (63, 64). Therefore, inhibition of SIRT1 inhibited the acetylation of Foxp3, which is essential for maintaining the suppressive function of Treg and the stable expression of Foxp3 (63, 64). Conversely, Mammalian Sterile 20-like Kinase 1(Mst1) is another serine/threonine kinase that plays an important role in immune cell development (65). In Tregs, the part of Mst1 is opposite to that of SIRT1. It is evident that Mst1 can interact with Foxp3 and enhances the activity of Foxp3 by stabilizing it *in vitro* and *in vivo (* 54). Interestingly, Mst1 could attenuate the SIRT1-dependent deacetylation of Foxp3 by impeding the interaction of SIRT1 and Foxp3 (47, 54). In our recent study, we have optimized that IκB Kinase α (IKKα), which PPARα (Peroxisome proliferator–activated receptor α) transcriptionally controls, regulates the Th17 development by modifying the Foxp3 expression. We found that overexpression of IKKα enhances Th17 development and leads to the proteasomal degradation of Foxp3 (66). Likewise, nucleus accumbens-associated protein-1 (NAC1) enhances the deacetylation of various proteins by interacting with HDACs (67). Nevertheless, targeted depletion of NAC1 accelerated the acetylation of Foxp3, whereas overexpression of NAC1 can decrease the acetylation of Foxp3 and may lead to the progression of autoimmune disease (68). Overall, it is well-understood that various proteins influence the acetylation of Foxp3, which interferes with the immune suppressive function of Tregs by affecting the stability of Foxp3.

## Glycosylation modification of Foxp3

Glycosylation-dependent PTMs significantly affect proteins’ activity, stability, conformation, folding, and distribution by attaching sugars (glycans and monosaccharides) to proteins through covalent binding (69). Changes in glycosylation are well associated with various cellular functions of T cells, including differentiation, development, activation, and apoptosis (70, 71). O-linked-N-acetylglucosaminylation (O-GlcNAcylation) is particular glycosylation that involves the attachment of a monosaccharide sugar, O-GlcNAc, to the serine or threonine residues (72, 73). During the process of O-GlcNAcylation, two enzymes, namely O-GlcNAc transferase (OGT) and O-GlcNAcase (OGA), catalyze the whole process by mediating the addition and removing the modification, respectively (74). Notably, O-GlcNAc is highly associated with Treg lineage stability, effector differentiation, and suppressive function (74). Mechanistically, several O-GlcNAcylation sites are present in Foxp3, which upon glycosylation mediates Foxp3 expression by regulating IL-2/STAT5 signaling pathway. Genetic ablation of OGT in Foxp3+ cells decreases Treg cell lineage stability, which can eventually elevate the severity of autoimmune diseases (74). Besides the direct role of O-GlcNAc on Foxp3, it can also influence the activity of Foxp3 binding partners. It was noted that c-Rel, a binding partner of Foxp3 (75), undergoes glycosylation, either by hyperglycemic- or chemically-induced O-GlcNAcylation, and reduces the binding of c-Rel with Foxp3 promoter; thus, and negatively regulates the immunosuppressive function of Tregs by reducing Foxp3 expression (76). Although a few studies have investigated the role of glycosylation in Tregs and Foxp3 regulation, the potential of O-GlcNAcylation targeted therapy in modulating the function of Tregs should be investigated to achieve anti-tumor and anti-autoimmune immunity (**Figure 2E**).

## Phosphorylation modification of Foxp3

Foxp3 also undergoes phosphorylation in the amino-terminal domain of Foxp3, which is modified at several sites (77) (**Figure 2B**). Specific protein kinase phosphorylation can regulate Treg development and stabilize the suppressive function of Tregs. Among these, cyclin-dependent kinases (CDK2) (78), proto-oncogene serine/threonine-protein kinase-1 (PIM1) (79), and PIM2 (77) kinase phosphorylate the Foxp3, thus negatively regulating it’s expression. Alternatively, PP1 (protein phosphatase 1) kinase negatively regulates Foxp3 by dephosphorylation (80), while CDK5 kinase could regulate Foxp3 complex protein by phosphorylation STAT3 (signal transducer and activator of transcription 3) (81).

Different mechanisms to regulate the phosphorylation of Foxp3 have been identified depending on the nature of protein kinases. For instance, CDK2 can bind to its partner cyclin E to phosphorylate Foxp3 at CDK motifs (S19, S88, T114, T175). This process could be inhibited by using the CDK inhibitor roscovitine (78). Similarly, PIM1 could negatively regulate Fopx3 expression by specific phosphorylation at Ser422. However, phosphorylation of Foxp3 at the Ser418 region could inhibit this phosphorylation at Ser422 (79). Interestingly, depletion of PIM1 increased Foxp3 binding activity and elevated the Foxp3-induced gene expression, such as CTLA-4, CD25, and GITR (Glucocorticoid-induced TNFR related protein), in human Tregs and decreased the expression of IL-2 gene, which ultimately improved the immunosuppression function of Tregs (79). In parallel to PIM1, PIM2, which also belongs to the PIM serine/threonine kinase family, was found to interact with Foxp3 in the human Tregs. Researchers found that the amino acid phosphorylation sites of PIM2 are located at Ser31 and Ser41. In addition, inhibition of PIM2 expression can increase the suppressive function of Treg cells and elevate the stabilization of Treg cell lineages (77).

Besides, CDK5 and PP1 regulate Foxp3 protein in different ways. As for PP1 can dephosphorylate Foxp3 at the Ser418 site in the C-terminal DNA-binding domain to decrease Treg-mediated suppressive function, particularly in the model of rheumatoid arthritis (82). Briefly, TNF-α produced in the inflamed synovium can induce the expression and enzymatic activity of PP1, which dephosphorylates Foxp3 and mediates a decrease in Treg suppressive function. Using a TNF-α specific antibody can restore the suppressive function of Treg in rheumatoid arthritis (82). In contrast, CDK5 phosphorylating Ser722 of STAT3, which promotes the transport of STAT3 to the nucleus and binds to the enhancer II region of Foxp3, increases the transcription and expression of Foxp3 and responds to the IL-6. Drug inhibition or targeted deletion of CDK5 can effectively attenuate Foxp3 expression and the suppressive ability of T cells (81). Thus, it can be suggested that phosphorylation of Foxp3 through various proteins can affect the function of Tregs.

## Ubiquitylation modification of Foxp3

The ubiquitination process involves the addition of a 3.5kDa ubiquitin protein to the target protein, which is driven by the action of ubiquitinates, including E1(ubiquitin-activating enzyme), E2(ubiquitin-conjugating enzyme), and E3 (ubiquitin ligase) (83). In the process of ubiquitination, adding a ubiquitin-protein to a target protein is monoubiquitylation; however, adding a chain of ubiquitin proteins to a target protein is polyubiquitylation (84, 85). Ubiquitination has been found to regulate various cellular and biological processes, including the cell cycle, transcriptional regulation, apoptosis, inflammatory response, and cell differentiation by targeting different proteins (86).

Ample evidence suggests the modification of Foxp3 by ubiquitination, which eventually regulates the Foxp3 expression and plays a vital role in maintaining the suppressive function and stability of Treg cells (87) (**Figure 2C**). For instance, lysine 48(K48)-linked polyubiquitylation often acts on target proteins for their proteasomal degradation, while K63-linked polyubiquitylation often acts in signal transduction cascades in cells (88). Meanwhile, polyubiquitylation is also associated with other lysine residues, such as K6, K11, K27, K29, and K33 (89–91). Our previous finding suggests that K63-linked ubiquitination plays an essential role in regulating the immunosuppression of Treg cells and the expression of Foxp3. Briefly, we found that TNF receptor-associated factor 6 (TRAF6) is ubiquitin-ligase, which regulates the K63 linked ubiquitination is used by the Foxp3 for its nuclear localization and its improvement in the transcription factor gene regulation activity in Treg by K63-linked ubiquitination (90). Conversely, another group of scientists found that TRAF6 interacts with Foxp3 by ubiquitination modification at lysine residue 262, which is necessary for the suppressive function of Treg, and they observed dysfunctional Tregs *in vivo* in TRAF6 depleted mice. Importantly, Treg-specific TRAF6 depleted cells attenuated tumor size and boosted anti-tumor immunity (92). Overall, these studies urge the potential of targeting TRAF6 to regulate Treg function.

Numerous stress factors, inflammatory cytokines, and lipids decrease the expression of Foxp3. It was found that this decrease in Foxp3 is modified by ubiquitination through E3 ubiquitin ligase Stub1 (STIP1 homology and U-box containing protein 1) (93). Mechanistically, the interaction between Stub1 and Foxp3 relies on the stress indicator protein Hsp70 (heat shock protein 70). Hsp70 recruits Stub1 and acts as a subunit of the Foxp3 complex, and the silencing of Stub1 expression enhances Treg suppressive function *in vitro* and *in vivo (* 93). Unlike the Stub1 protein, MALAT1 (metastasis associated lung adenocarcinoma transcript 1) can increase Foxp3 expression through another mechanism. MALAT1 generally covers the Stub1 interaction region of Foxp3 with modified proteins so that it cannot be modified by Stub1 ubiquitination, resulting in stable expression of Foxp3 (94). Apart from that, MALAT1 also plays a key role in the post-transcriptional modification of Foxp3, which affects the GINS1 (GINS Complex Subunit 1) transcription and ultimately leads to the progression of NSCLC (94).

Loss of Foxp3 expression is linked with the pathogenesis of multiple autoimmune diseases, including type 1 diabetes in NOD mice (95). This was further testified by Matthew et al. in a Foxp3^gfp^ reporter mouse on NOD background mice. They illustrated that nTreg development and function were not significantly different *in vitro* in Foxp3^gfp^ NOD and C57BL/6 mice, as autoimmune diabetes on an NOD background was accelerated (96). In contrast, Treg cell development was reduced under inflammation or induction by TGF-β. This was because Foxp3^gfp^ could not interact with Tip60, HDAC7, and EOS, which reduced the stability of Foxp3. Correspondingly, the enhanced K48-linked polyubiquitination resulted in Foxp3 degradation, decreased Treg development, and immunosuppressive function (96–98). Collectively, it can be reported that the ubiquitylation of Foxp3 plays a significant role in the stability and function of Foxp3.

## Methylation modification of Foxp3

In addition to the PTMs mentioned above, methylation of transcription factors or co-stimulatory factors is also important for the regulation of gene expression (99). Altered methylation of Foxp3 is linked with the onset of multiple diseases (100, 101) (**Figure 2D**). The main sites of methylation modification are arginine and lysine residues (102). Multiple pieces of evidence suggest that protein arginine methyltransferase (PRMT) family members PRMT1 and PRMT5 regulate the suppressive function of Treg cells by introducing methyl group to regulate Foxp3 transcription activity and exert a vital role in autoimmune and cancer diseases (103–106).

A deeper understanding revealed that the Foxp3 transcription factor is methylated at arginine residues at 48 and 51 via PRMT1 (105). Methylation at these two sites promotes Treg cell-mediated suppressive function. The use of MS023 (type I protein arginine methyltransferase (PRMT) inhibitor) confers Th1-like gene expression profiles to Foxp3-expressing T cells (105). Importantly, PRMT1 also plays a crucial role in the differentiation between Th17 and Treg. PRMT1 is associated with RORγt and regulates Th17 differentiation. It was found that overexpression of PRMT1 effectively promotes the Th17 expansion, but inhibition or deletion of PRMT1 reduced the Th17 differentiation, which ultimately leads to an increased Foxp3^+^ Tregs population (107). Therefore, it can be assumed that the methylation of transcription factors can effectively regulate gene expression through methyltransferases.

In addition, the mass spectrometric analysis showed that FOXP3 can be di-methylated at positions R27, R51, and R146 by PRMT5. Whereas arginine(R)51 is mutated to Lysine(K), leading to the loss of suppressive function in human CD4 T cells (106). These PRMT5 knockout mice can develop severe scurfy-like autoimmune diseases in mice by showing an impaired number of Treg cells in the spleen, while no change was observed in the Tregs from peripheral lymph nodes (106). However, the peripheral Treg with PMRT5 knockout exhibited decreased suppressive function (106). DS-437 (Pharmacological inhibitors for PMRT5) can enhance anti-erbB2/neu monoclonal antibody targeted therapy (106). In contrast, Zheng et al. described that PRMT5 inhibition in mice can resolve autoimmune diseases by elevating the number of Treg cells (108). These studies conclude that PRMT1 and PRMT5 are necessary for post-transcriptional methylation of Foxp3. Meanwhile, further studies are required to fully understand the role of PRMTs in PTMs and the function of Foxp3. Overall, these studies urge that targeting the methyltransferases may serve as potential therapeutic targets to achieve Treg-mediated immunotherapy.

## Targeting Treg as a potential immunotherapy

Targeted deletion of Tregs in tumors is a fascinating and promising treatment; however, deleting Tregs can cause autoimmune diseases and affect the effectiveness of treatment. Thus, researchers are interested in developing therapies that selectively target the immunosuppressive Tregs in tumors without disrupting the normal inflammatory response, effector T cells, or causing autoimmune diseases. Since CD25 is a highly expressed surface marker of Treg cells, targeting CD25 to delete Treg cells is a suitable choice. In mice, the anti-CD25 antibody can effectively delete Treg *in vivo* and enhance anti-tumor immunity (109, 110). An earlier study in clinics demonstrated that using an anti-CD25 antibody along with daclizumab to delete Treg in breast cancer patients successively eliminates the CD4^+^CD25^+^Foxp3^+^ Treg cells in peripheral blood. Using daclizumab combined with a specific tumor antigen vaccine can effectively activate the production of toxic T lymphocytes (111). However, the effector T cells also express CD25 and require IL-2, and the use of daclizumab also affects their killing function.

Targeting CCR8 has also been presented as an effective therapy to target the Tregs. It has been illustrated that usage of anti-CCR8 alone or in combination with other immune checkpoint inhibitors promotes the anti-tumor immune response by depleting the intratumoral Tregs (112–115). Meanwhile, Sugiyama et al. reported that targeting CCR4 in Tregs by using anti-CCR4 can selectively deplete the Tregs and augment the response of tumor-specific CD8+ T cells, thereby improving the anti-tumor immune response (116). Another attractive therapeutic target site is CTLA-4. Technically, the competitive binding of CTLA-4 on CTLs with CD80/CD86 on APCs bypasses CD28 on APCs and suppresses hyperactivated cytotoxic T cells (117). But using immune checkpoint mAbs, ipilimumab, and tremelimumab (118), avoids a decline in immune responses, increases the number of activated T cells, and promotes T cell–tumor cell interactions (119). Some studies have found that ipilimumab can induce Fc-mediated antibody-dependent cytotoxicity (ADCC)-mediated Treg cell reduction in Pancreatic cancer due to the infiltration of FcγR-dense myeloid cells in the tumor, which increases the sensitivity to CTLA-4 mAbs (120, 121). In contrast, tremelimumab does not bind to the FcγR of human leukocytes to release the IL-2 cytokine, avoiding cytokine release syndrome. However, the antibody can enhance IL-2 cytokine production by T cells in healthy individuals or patients with tumors, including ovarian, renal, prostate, and rectal cancers (122). Programmed cell death protein 1 (PD-1), an inhibitory receptor for tumor-specific T cells, can bind to the PD-L1 ligand produced by tumor cells to inhibit T cell effector function. CTLA-4 and PD-1 inhibitory immune checkpoints can suppress the activation of effector T cells. Interestingly, combined treatment with CTLA-4 mAbs (Ipilimumab) and PD-1 (nivolumab) mAbs can kill tumor cells to the greatest extent and delay the occurrence and development of tumors, including metastatic melanoma, metastatic renal cell carcinoma, colorectal cancer with MSI-H and MMR aberrations (123–125). Interestingly, it has been validated that use of anti–PD-1 decreases the tumor infiltrating Tregs (126). Meanwhile, the use of anti-CTLA-4 doesn’t influence the population of Foxp3+ Tregs in the tumors (127). These new findings suggest that CTLA-4 and PD-1 may serve as novel target for the future in different tumor therapy.

In addition to the receptors mentioned above, numerous other molecules are also expressed on the surface of Treg cells, which may serve as target sites for Treg deletion. GITR belongs to glucocorticoid-induced TNF-receptor family-related protein, which is highly expressed on the surface of naïve Treg cells and intermediately expressed on the surface of naïve CD4/CD8, naïve myeloid cells. Some researchers have shown that the anti-GITR monoclonal antibody DTA-1 can effectively destabilize Treg, leading to the loss of Foxp3 expression, which is essential for maintaining Treg suppressive function (128–131). However, another study reported that GITR-dependent therapies are context-dependent as GITR modulates the balance between effector CD4+ T cells and Tregs by elevating their proliferation of both populations in parallel (132, 133). OX40 is another co-stimulatory molecule of the TNF receptor family, expressed in activated CD4/CD8 T cells and constitutively expressed on most Tregs (134, 135). It appears to act on Treg in a different mechanism compared to GITR. Conjugation of OX40 to agonist OX40-specific reagents can effectively enhance anti-tumor immunity and regulate the generation of effector and memory T cells (136, 137). Using agonist OX40 can effectively delete intertumoral Treg while combining chemotherapy can directly modulate the inhibitory effect of intertumoral Treg (138, 139). Some preclinical experiments have shown that OX40 ligation can weaken the suppressive function of Treg. Thus, GITR and OX40 are novel immunotherapy targets currently being tested in clinical studies (140).

Similarly, WO2017011559A1 (anti-CCL20 Abs) can bind to CCL20 and inhibit the interaction between CCR6 and CCL20, thereby mediating the inhibition of Treg/Th17 recruitment to the tumors, which can inhibit cancer stem cell activity and the tumorigenesis (141). However, inhibiting CCR6 will also affect the recruitment of other anti-tumor cells or lead to the onset of autoimmune diseases *in vivo* because Tregs are also recruited to other tissues by CCR6 to play an immunosuppressive role (142). Alternatively, research studies have shown the detrimental effects of Tregs-targeted immunotherapy on the body and other organs. Therefore, more efforts should be put into achieving Treg-targeted therapy with minimal adverse effects and prolonging the survival of the patients.

## Targeting PTMs in Tregs as a potential therapeutic strategy

Meanwhile, we also summarized the mechanisms defining the suppressive function of Tregs, particularly through PTMs, such as phosphorylation, glycosylation, ubiquitination, acetylation, and methylation. We demonstrated that these PTMs might contribute to fine-tuning Treg function, stability, differentiation, and lineage development. Considering the diverse links between Tregs and TME regulation, various potential drugs targeting PTMs have demonstrated their efficacy in *in-vivo* and clinical studies, which influence the function and differentiation of Tregs. For instance, pharmacological downregulation of PRMT5 by using DS-437 can decrease the overall population of Tregs by significantly reducing Foxp3 methylation (106). Conversely, a new study investigating the impact of D1ManPrup3, a synthetic glycodendropeptides, on the Treg-dependent immune tolerance and desensitization suggests that the use of D1ManPrup3 may lead to methylation changes in Foxp3 and alternatively influence the function of Tregs (143). Meanwhile, a recently recognized drug Qi-Dong-Huo-Xue-Yin (QD), which is a traditional Chinese medicine widely used in treating COVID-19 patients, showed a positive role in the development of Tregs by enlightening the Foxp3 acetylation in CD4+ T cells (144).

Although various studies have identified the drugs involved in diminishing the population of Tregs in tumors, how to advance Treg-specific targeting remains challenging. In recent years, strategies to target Tregs systematically or locally by using ligand-directed toxins or monoclonal antibodies have been adopted (145). Nevertheless, not all healthcare problems respond effectively to such treatment methods, particularly autoimmune disorders and other inflammation-related disorders. In order to target Tregs specifically, similar to other cell-type-specific medications, a computational tool and platform must be developed. The most efficient initial step in accomplishing this is to avoid tackling the isoforms of Foxp3. In clinics, four HDAC inhibitors are used to achieve anti-tumor responses in the USA (59). For instance, the usage of Trichostain A (TSA), a class I/II HDAC inhibitor, enhanced the proportion of Treg cells and their immunosuppressive activity (146), which was mainly associated with the inhibition of class IIb HDACs (59). Since HDAC-6 and -10 are members of class IIb HDAC and have been shown to have more significant roles in Treg activities *in vivo*, an inhibitor specifically designed to target HDAC6 may eventually provide the best immunotherapeutic response (59, 147). In parallel, targeting E3 ligases is a crucial additional strategy for achieving Treg-specific targeted therapy (148). The use of nanoparticles to specifically target Tregs is also fascinating. Recent investigations have employed uniquely designed particles to deliver antigens or medications to Treg cells (149–152), implying that targeting PTMs through small-molecule inhibitors may also be employed in developing new therapeutic strategies.

A recently developed approach, Proteolysis Targeting Chimaeras (PROTACs), represents an innovative technique in the realm of pharmacology, offering a solution to the challenge of targeting proteins that were previously considered hard to target. While conventional medications typically bind to target proteins, PROTACs function as molecular orchestrators, initiating the specific degradation of particular proteins within cells (153). Comprising a ligand for the protein of interest (POI), a ligand for an E3 ubiquitin ligase, and a connecting linker, PROTACs instigate a cascade of events (153, 154). They facilitate rapid ubiquitination and subsequent proteasomal degradation of the target protein by effectively bridging the POI and the E3 ligase, bringing them into close proximity (154, 155). In a recent study, anti-apoptotic B-cell lymphoma extra-large (BCL-X_L_) was identified as a potential therapeutic target within tumor-infiltrating Tregs. The authors used a specialized PROTAC molecule, which induced the BCL-X_L_ degradation, leading to Treg apoptosis within the tumor microenvironment, suggesting a novel strategy to boost anti-tumor immune responses (156). Similarly, a previous study uncovered a process involving the degradation of the Foxp3 protein in response to LPS stimuli facilitated by the E3 ligase STUB1 and the heat shock protein HSP70. This degradation effectively impairs the suppressive function of Tregs (93). Although studies using the PROTACs in targeting Tregs are very limited, we believe that this innovative approach holds great promise in drug development, offering a precise means of modulating cellular processes by selectively eliminating Foxp3, with potential in anti-tumor immunity.

## Conclusion and perspectives

Over the past decades, researchers have been working to understand the suppressive function of Tregs in the TME and the factors regulating these suppressive functions of Tregs and the Foxp3 expression. So far, the complex interaction between Treg and various intertumoral cells hasn’t been completely studied. It is still unknown which Treg inhibitory mechanism plays the main role or whether multiple inhibitory mechanisms work together. Here, we have also summarized the role of various factors in mitigating the Treg accumulation in tumors and the immunosuppressive mechanisms of Treg. Finally, we recapitulated the advances in Treg-targeted immunotherapy including CD25, CTLA-4, PD-1, OX-4O, GITR, chemokines receptors, and ligands.

This review also summarizes various PTMs that are vital in regulating Foxp3 protein stability and immunosuppressive functions. These PTMs, which can occur at different sites, help in initiating, terminating, or fine-tuning the Treg-dependent immune responses. Importantly, protein complexes create a dynamic network of PTMs that govern the function of Foxp3 by altering the physical and chemical properties, affecting its conformation, function, and interactions with Foxp3 transcription factor complexes. Additionally, Foxp3 PTMs may play a role in context-specific gene regulation by altering the stability of Foxp3 transcription factor complexes. However, multiple key challenges persist in targeting PTMs in achieving anti-tumor immunity. The hurdles and uncertainties include: (1) how metabolic-linked PTMs modulate Treg function; (2) hard to forecast the effect of specific modifications on the development and function of Tregs; (3) the effects of various endogenous factors, such as microbiota in TME, on the Treg function is not known; (4) the factors which influence the role of Foxp3 transcription factor complexes between transcriptional repressor and activator are not fully known; (5) the mechanism by which transcription factor complexes lead to the PTMs of Foxp3 are not entirely understood; (6) an efficient and effective delivery and evaluation of drugs targeting PTMs in Tregs is necessary to determine the potential and cost-effectiveness of Treg-targeted anticancer treatment.

The significance of this discovery lies in the critical role of Tregs in maintaining immune balance, where both their reduction and elevation can, respectively, contribute to autoimmune diseases and cancers. Consequently, developing drugs that target the interaction of Foxp3 with its binding partners, either restrict or enhance their interaction, holds promise for the development of Treg-specific treatments with potential applications in immune-related disorders and cancer therapy. Overall, understanding the complex signaling complexes that are involved in crosstalk between Foxp3 and several PTMs, and clarifying the association of these complex networks will highlight the Treg biology in immune health and diseases. Also, this PTM machinery will help us identify the novel targets for developing Treg-targeted drugs to achieve anti-tumor and anti-autoimmune immune responses.

## Author contributions

FR: Conceptualization, Writing – original draft, Writing – review & editing. ZH: Writing – original draft. FP: Funding acquisition, Supervision, Writing – review & editing.
